# Differential Requirement for the Cell Wall Integrity Sensor Wsc1p in Diploids Versus Haploids

**DOI:** 10.3390/jof7121049

**Published:** 2021-12-08

**Authors:** Allison E. Hall, Miriam Lisci, Mark D. Rose

**Affiliations:** 1 Department of Molecular Biology, Princeton University, Princeton, NJ 08544, USA; Allison.Hall@nyulangone.org (A.E.H.); Ml793@cam.ac.uk (M.L.); 2 Helen L. and Martin S. Kimmel Center for Biology and Medicine, Skirball Institute of Biomolecular Medicine, New York, NY 10016, USA; 3 Cambridge Institute for Medical Research, University of Cambridge, Cambridge Biomedical Campus, Cambridge CB2 0XY, UK; 4 Department of Biology, Georgetown University, Washington, DC 20057, USA

**Keywords:** cell wall integrity, conjugation, ploidy, yeast, lysis, protein kinase C

## Abstract

The primary role of the Cell Wall Integrity Pathway (CWI) in *Saccharomyces cerevisiae* is monitoring the state of the cell wall in response to general life cycle stresses (growth and mating) and imposed stresses (temperature changes and chemicals). Of the five mechanosensor proteins monitoring cell wall stress, Wsc1p and Mid2p are the most important. We find that *WSC1* has a stringent requirement in zygotes and diploids, unlike haploids, and differing from *MID2*’s role in shmoos. Diploids lacking *WSC1* die frequently, independent of mating type. Death is due to loss of cell wall and plasma membrane integrity, which is suppressed by osmotic support. Overexpression of several CWI pathway components suppress *wsc1*∆ zygotic death, including *WSC2*, *WSC3*, and *BEM2,* as well as the Rho-GAPS, *BEM3* and *RGD2*. Microscopic observations and suppression by *BEM2* and *BEM3* suggest that *wsc1*∆ zygotes die during bud emergence. Downstream in the CWI pathway, overexpression of a hyperactive protein kinase C (Pkc1p-R398P) causes growth arrest, and blocks the pheromone response. With moderate levels of Pkc1p-R398P, cells form zygotes and the *wsc1*∆ defect is suppressed. This work highlights functional differences in the requirement for Wsc1p in diploids Versus haploids and between Mid2p and Wsc1p during mating.

## 1. Introduction

The budding yeast, *Saccharomyces cerevisiae*, is a single-celled organism that lives in a relatively harsh environment in the wild, undergoing stresses due to changes in osmotic pressure and temperature. To survive in this environment, yeast have evolved a robust cell wall that protects against rapid osmotic changes and mechanical stresses, while providing support for shape changes during budding and polarized growth in response to pheromone [1,2].

**Figure 1:**
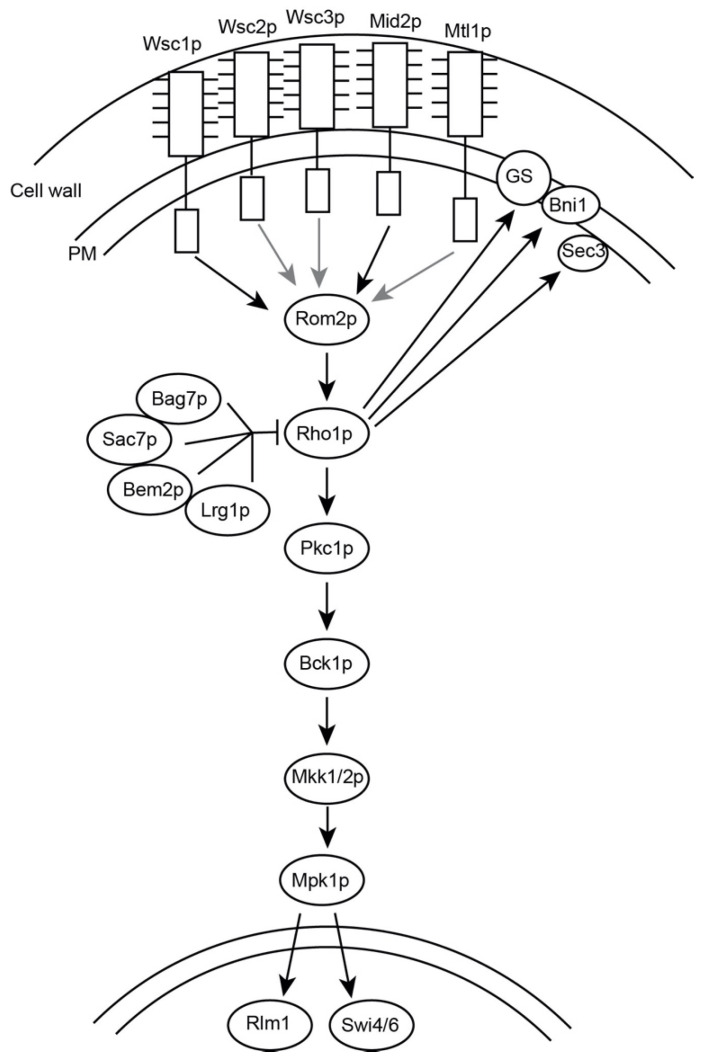
Overview of the Cell Wall Integrity Pathway. The CWI transmits signals through five transmembrane proteins, Wsc1p, Wsc2, Wsc3, Mid2p and Mtl1p. These proteins are anchored in the plasma membrane (PM) and extend into the cell wall. The transmembrane proteins signal through Rom2p to activate Rho1p. Rho1p is responsible for the activation of the 1,3-β-glucan synthase (GS), a formin (Bni1p), Sec3p, and Pkc1p. Rho1p is acted upon by four Rho-GAPs, Sac7p, Bag7, Lrg1, and Bem2p. The CWI pathway is a MAP kinase cascade that culminates in the phosphorylation of Mpk1p, which activates transcription factors Rlm1p and SBF (Swi4p/Swi6p) to activate genes involved in cell cycle regulation and cell wall biogenesis. (Adapted from Levin, 2005 and Levin, 2011).

Stresses to the cell wall are primarily detected by the cell wall integrity (CWI) pathway [3,4,5]. The CWI pathway is a signaling cascade that is regulated by five transmembrane sensor proteins that extend into the cell wall (Wsc1-3p, Mid2p, and Mtl1p) and ultimately regulate a series of MAP kinases [6,7,8,9] (Figure 1). Wsc1p and Mid2p appear to be the most important of the transmembrane proteins; *wsc1*∆ *mid2*∆ cells die without osmotic support (1 M sorbitol) [6,9] whereas cells containing only Wsc1p and Mid2p grow like wildtype [10]. The transmembrane proteins interact with Rom2p, a guanosine-nucleotide exchange factor (GEF) for Rho1p, a GTPase and master regulator of the CWI pathway [8]. Rho1p plays a role in regulating cell wall deposition through the 1,3-β-glucan synthase (GS) complex, made up of Fks1p and Fks2p [11,12,13]. Rho1p also binds to, and activates, the sole protein kinase C in yeast, Pkc1p, and has effects on the actin cytoskeleton and polarized secretion [14,15,16,17,18].

Rho1p cycles between GTP and GDP bound states, which are regulated by a set of GTPase-activating proteins (GAPs) and GEFs. Rom1p and Rom2p are the activating GEFs that respond to signals from the transmembrane proteins in the CWI pathway [17]. Four Rho-GAPs act on Rho1p: Bag7p, Sac7p, Lrg1p, and Bem2p [1,19,20,21,22,23] (Figure 1). The Rho-GAPs act on Rho1p in a target specific manner. Lrg1p primarily functions to control GS, Bem2p and Sac7p collaborate to down-regulate the CWI pathway, and Bag7p and Sac7p act on the actin cytoskeleton [19,20,22,23,24]. GTP-bound Rho1p associates with and activates Pkc1p [14,16]. 

Rho1p activation of Pkc1p allows stimulation of a downstream MAP kinase cascade, eventually culminating in the phosphorylation and activation of Mpk1p [14,25,26,27]. Mpk1p translocates to the nucleus, where it activates transcription of genes involved in cell wall biogenesis and the cell cycle [5]. Deletion of Pkc1p causes cell lysis at all temperatures; loss of function of any protein downstream of Pkc1p leads to cell lysis at elevated temperature. Lysis is suppressed by high osmolarity (1 M sorbitol), indicating that death is due to defects in the cell wall [28,29,30,31]. 

Like *pkc1*∆, deletion of *WSC1* leads to cell lysis, but only at elevated temperatures. The *wsc1*∆ cells also show increased sensitivity to certain drugs, such as caffeine and Caspofungin [32,33]. Deletion of *MID2*, on the other hand, causes pheromone sensitivity and sensitivity to Calcofluor White [6,10]. Both Wsc1p and Mid2p are thought to act as mechanosensors, presumably to sense changes in the cell wall for rapid response [34,35]. However, their different roles in mitotic growth and mating and the differential drug sensitivity suggest that the proteins have similar, but not completely overlapping, functions. Mid2p and Wsc1p localize to distinct microdomains within the plasma membrane, perhaps allowing for their ability to sense unique perturbations on the cell wall [36]. Wsc1p localizes to sites of polarized growth in both mitotic and mating cells [3,37,38]. While much is known about how Wsc1p contributes to temperature sensitivity in haploid cells, [39,40] its function during other stresses and life cycle stages remains less clear.

The CWI pathway is well known for its role in sensing and responding to stresses on the cell wall. Previous work has also indicated a role for the CWI pathway during mating, a situation in which cell wall degradation is actually promoted. A hyperactive allele of Pkc1p (PKC1-R398P) causes a block to cell fusion [41], and we have previously shown that *MID2* and *PKC1* negatively regulate cell fusion, which is relieved upon cell-cell contact [10]. Given the similarities between Mid2p and Wsc1p, do both proteins regulate cell fusion? During mating, zygote formation, and subsequent budding, there are dramatic changes to the cell wall and the shape of the cell. To mate, haploid cells arrest in the cell cycle and polarize growth toward one another along a pheromone gradient, forming a pear-shaped cell called a shmoo. After the mating partners come into contact they must degrade their cell wall at the zone of cell fusion (ZCF) to allow for plasma membrane fusion, cytoplasmic mixing, and nuclear fusion [42,43,44]. After fusion, zygotes reenter the cell cycle and usually bud from the neck connecting the two mating partners [45,46]. Each of these steps requires changes in cell shape and cell wall remodeling, which are likely sensed and regulated by the CWI pathway. 

During a screen for mating phenotypes caused by loss of function mutations of the CWI pathway transmembrane proteins, we found that *WSC1* has a zygote/diploid specific role. Whereas *wsc1*∆ causes temperature sensitive growth in haploids, *WSC1* is necessary for diploid survival. The requirement is conferred by cell ploidy and not by mating type. Thus, Mid2p and Wsc1p have distinct roles; Mid2p is necessary in shmoos, whereas Wsc1p is critically important in diploids, providing a possible reason for why both proteins have been retained throughout evolution. 

## 2. Materials and Methods

### 2.1. General Yeast Techniques, Strain and Plasmid Construction

Yeast media, general methods, and transformations were performed as described previously [47] with minor modifications. Strains and plasmids are listed below. Deletion strains were either created via PCR amplification of selective markers and homologous recombination at the locus of interest, or via sporulation and tetrad dissection. Where indicated cells were grown in YEPD supplemented with 1 M sorbitol. 

All strains were grown at 30 °C. For pheromone induction experiments, early exponential cells growing in selective media were treated for 90 min (unless otherwise specified) with synthetic α-factor (Department of Molecular Biology Syn/Seq Facility, Princeton University, Princeton, NJ, USA) added to a final concentration of 10 μg/mL.

### 2.2. Yeast Mating Assays

Limited plate mating assays and quantitative filter-matings were performed as described previously with minor alterations [48]. Briefly, limited plate mating assays used a lawn of the *MAT***a** strain grown on rich media plates, and patches of the *MAT*α strains, grown on rich media or selective media for strains containing plasmids. The strains were replica plated together onto rich media, allowed to mate for 3 h at 30 °C, and then replica plated onto media selective for diploids. Mating efficiency was assessed after 2 days of growth at 30 °C. For *wsc1*∆ mating at low temperature, plates were grown, mated, and incubated at 23 °C. Matings were performed for 3 or 5 h and mating efficiency was assessed after 3 days of growth to account for slowed growth rate.

Quantitative filter-matings were performed by mixing early exponential *MAT***a** cells with *MAT*α cells at a 1:1 ratio of optical density units to reach a total of ~1 × 10^7^ cells/mL. The cells were mixed together, concentrated on 25 mm 0.45 μm nitrocellulose filter disks (Millipore Corporation, Burlington, MA, USA), and incubated on rich media plates for 2.5–3 h at 30 °C.

### 2.3. High Copy Suppression of wsc1∆

A YEp13-based yeast genomic DNA library [49] was transformed into *MAT* α *wsc1*∆ strain. Approximately 24,000 transformants were mated to a *MAT***a** lawn (MY14305) as described above. Plasmids showing suppression were recovered from the cells [47] transformed into MY14306, and retested. DNA sequencing was used to identify the genes carried on the suppressing plasmids.

### 2.4. Microscopy

All images were acquired at 23 °C using a deconvolution microscopy system (DeltaVision; Applied Precision, LLC) equipped with an inverted microscope (TE200; Nikon) and a 100x objective with numerical aperture of 1.4. Deconvolution and image analysis were performed using Precision softWoRx and ImageJ (National Institutes of Health).

For imaging with propidium iodide, cells were prepared as described above and imaged after three hours in pheromone. Cells were spun down and resuspended in PBS, propidium iodide was added to a final concentration of 20 µm, and cells were incubated for 15 min at 23 °C in the dark prior to imaging.

Live/Dead Yeast Viability Kit (Invitrogen) was used according to protocol. Cells prepared as above after three hours in pheromone. Final concentration of 15 µm FUN-1 and 25 µm Calcofluor white were used prior to imaging.

Microscopic assays of FM4-64 stained mating mixtures and pheromone induced cells were performed as described previously [50]. Pheromone induced cells were prepared as described and then resuspended in 1 mL of TAF buffer (20mM Tris-HCl, 20mM NaN_3_, 20mM NaF in water) and kept on ice. FM4-64 (Molecular Probes/Invitrogen) was added to cells to a final concentration of 4 μM and stained shmoos were imaged as above. For fusion assays, an equal OD_600_ (0.5) of each mating type in log phase was mixed, concentrated on 25 mm 0.45 μm nitrocellulose filter disks (Millipore Corporation), and incubated on rich media plates for 2.5–3 h at 30 °C. FM4-64 staining was as above.

Live imaging was performed by mixing 0.02 OD_600_ of *wsc1*∆ *MAT***a** and *wsc1*∆ *MAT*α cells on a 2% agarose pad and imaged at 23 °C. Images were taken at two-minute intervals using the DeltaVision system described above.

Chi-squared statistical tests were used to determine *p*-values for microscopy data.

### 2.5. WSC1 Plasmid Loss and Weaning Experiments

Diploid cells obtained as described above from plate mating assays were grown in YEPD and maintained in log phase, without being allowed to reach saturation. Log phase cultures were allowed to grow for eight generations. The same population of cells were allowed to reach saturation and treated as follows. Cells were plated on either YEPD or 5-FOA plates and allowed to grow for 2 days at 30 °C, at which time colony forming units were counted and plasmid loss was determined as colonies on YEPD/colonies on FOA.

For sorbitol weaning experiments, *WSC1*/*wsc1*∆ or *wsc1*∆/*wsc1*∆ diploid cells were streaked for single colonies on FOA + 1 M sorbitol plates to ensure the loss of URA plasmids (empty vector or *WSC1* covering plasmid). Cells were subsequently maintained in log phase in YEPD + 1 M sorbitol media and the osmolarity of the media was reduced by 0.2 M every two hours to allow for one doubling at each condition. Cells were allowed to double once in 0M sorbitol media. Another population of cells were maintained in YEPD media for the duration of the experiment. Cells were plated on YEPD or YEPD + 1 M sorbitol plates and allowed to grow for 2 days at 30 °C. Colony forming units were counted and plotted as cells on YEPD/cells on YEPD + 1 M sorbitol. One-tailed Student’s *t*-tests were used to determine *p*-values for plasmid loss experiments.

### 2.6. Western Blotting

Cell extracts were prepared from 2 OD_600_ of each sample which had been treated with β-estradiol (for P_Z3EV_ *PKC1*) as previously described [50]. Where noted, samples were grown in YEPD or YEPD + 1 M sorbitol. Samples were lysed using NaOH/β-mercaptoethanol (1.85 M NaOH and 7.4% β-mercaptoethanol) and 50% trichloroacetic acid. Samples were run on a 10% polyacrylamide gel at 200 V for 2 h and transferred onto a nitrocellulose membrane at 100 V for 1 h. Detection of Pkc1p was done with an anti-Pkc1p antibody (Santa Cruz Biotechnology) used at 1:1000. Kar2p, used as a loading control, was detected with a custom anti-Kar2p antibody [51] used at 1:1000. HRP-conjugated anti-mouse secondary antibodies (Santa Cruz) were used at 1:2500.

## 3. Results

### 3.1. WSC1 Is Required for Diploid Survival 

**Figure 2:**
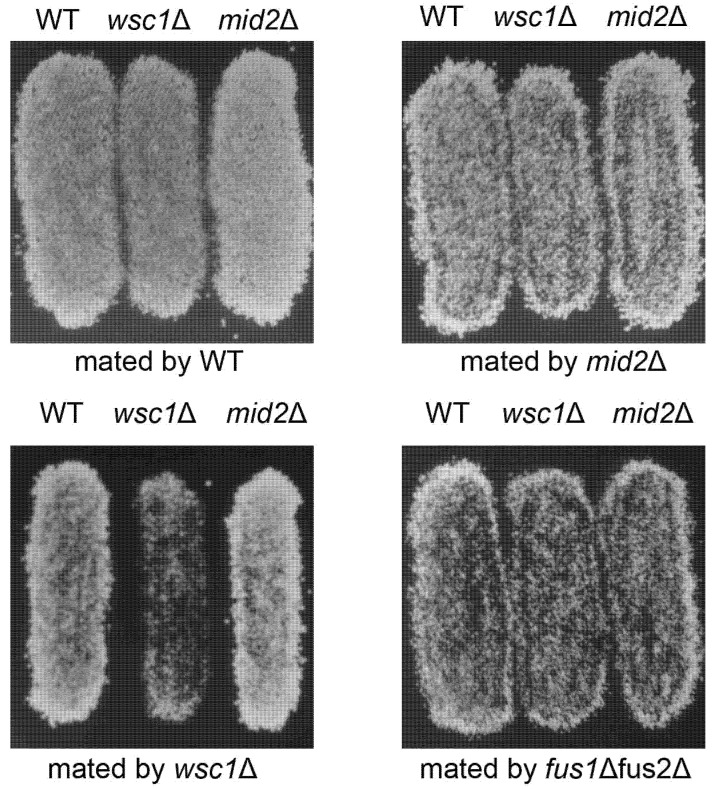
Bilateral loss of *WSC1* causes a defect in diploid formation. *MAT***a** patches of WT (MY8092), *wsc1*Δ (MY14305), and *mid2*Δ (MY14673) were mated to lawns of *MAT*α, WT (MY8093), *wsc1*Δ (MY14306), *mid2*Δ (MY14674) and *fus1*Δ *fus2*Δ (JY429). Cells were mated for 3 h at 30 °C and replica plated onto diploid selective media. In each case the images are of patches of cells growing on 8.5 cm petri dishes; the image panels are 4.0 cm in width.

Wsc1p and Mid2p are thought to act as mechanosensors, detecting changes in the cell wall and transducing signals to activate the CWI pathway [34,35]. Given the evidence that the CWI pathway plays a role in cell fusion, we tested deletions of the five transmembrane sensor proteins, by mating deletions by themselves, to *fus1*∆ *fus2*∆, or to wild type strains, to determine if their loss caused a mating defect. Cells exhibiting a mating defect do not form diploids efficiently when mated to *fus1*∆ *fus2*∆ strains [42]; we thus used this strain to initially determine if *wsc1*∆ cells showed a mating defect. Only the *wsc1*∆ mutant showed a strong apparent mating defect, specifically when mated to another *wsc1*∆ strain (bilateral mating), but not when mated to a *WSC1* wild type (Figure 2). In contrast, the *mid2*∆ mutation did not cause a detectable mating defect, whether mutants were mated to *mid2*∆, *wsc1*∆, or *fus1*∆*fus2*∆ mutants or the wild type (Figure 2). Deletions of the remaining three genes encoding transmembrane sensor proteins (*WSC2*, *WSC3* and *MTL1*) caused no apparent defects in mating. 

**Figure 3:**
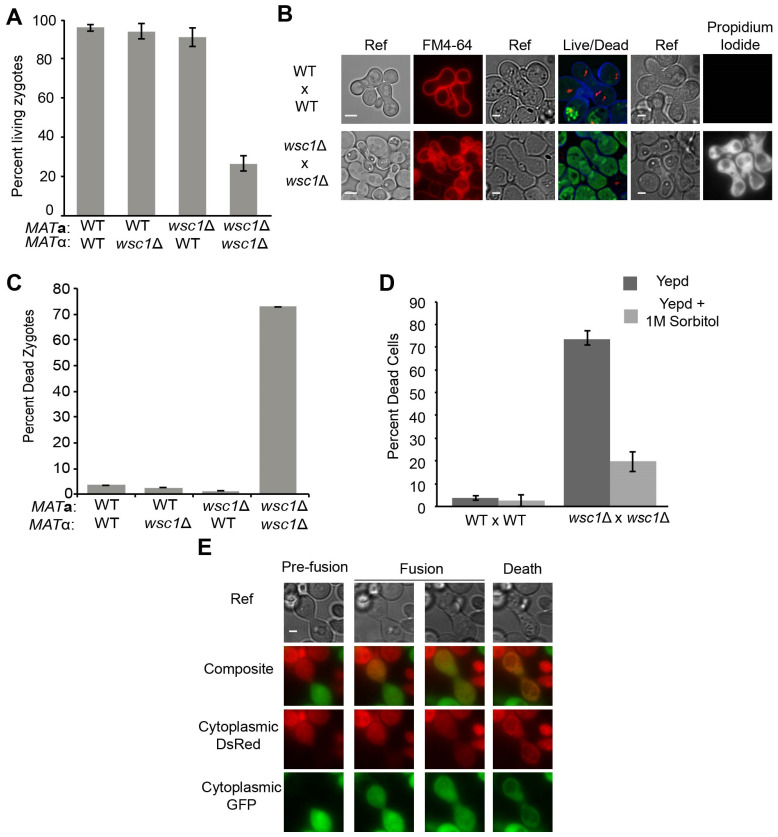
*wsc1*Δ causes zygote death. (**A**) Growth of zygotes formed WT and *wsc1*Δ partners. WT x WT (MY8092 xMY8093) or *wsc1*Δ x *wsc1*Δ (MY14305 xMY14306) matings were performed for 2 h. Individual zygotes were physically moved to new positions using a micromanipulator and dissecting microscope and allowed to grow for 2 days at 30 °C. Live zygotes grew to form individual colonies. n = ≥100 zygotes for each. Error bars represent the standard error. (**B**,**C**) *wsc1*Δ x *wsc1*Δ zygotes are not metabolically active and lose membrane integrity. (**B**) WT x WT (MY8092 x MY8093) or *wsc1*Δ x *wsc1*Δ (MY14305 x MY14306) cells were mated for 3 h at 30 °C. Cells were stained with FM4-64, Live/Dead staining (FUN 1 and Calcofluor White) or propidium iodide. Scale bar = 2 µm. (**C**) Microscopic quantification of zygote viability between WT and *wsc1*Δ. Filter matings of WT x WT (MY8092 x MY8093), WT *MAT***a** x *wsc1*Δ *MAT*α (MY8092 x MY14306), WT *MAT*α x *wsc1*Δ *MAT***a** (MY8093 x MY14305) or *wsc1*Δ x *wsc1*Δ (MY14305 x MY 14306) were performed for 3 h at 30 °C. Zygotes were resuspended in TAF buffer, stained with FM4-64 and imaged. Brightly stained zygotes were counted as dead. n > 400. Error bars represent the standard error. (**D**) Osmotic support suppresses *wsc1*Δ x *wsc1*Δ zygote death. WT (MY8092, MY8093) and *wsc1*Δ (MY14305, MY14306) cells were grown in YEPD or YEPD + 1 M sorbitol, mated for 3 h at 30 °C on the media they were grown in, stained with FM4-64 and imaged. n > 150. Error bars represent the standard error. (**E**) Live imaging of *wsc1*Δ x *wsc1*Δ mating. *wsc1*Δ *MAT***a** (MY15101) cells transformed with an integrating plasmid containing cytoplasmic GFP (MR5909) were mated to *wsc1*Δ *MAT*α (MY15102) cells transformed with an integrating plasmid containing cytoplasmic DsRed (MR5908). Cells were mixed and imaged at 2-min intervals. Images show cells prior to fusion and cytoplasmic mixing, after fusion when both markers have transferred, and the change in fluorescence as zygotes die. Scale bar = 2 µm. Chi-squared statistical tests were used to determine significance.

To determine the cause of the defect in the bilateral *wsc1*∆ mating, we performed a “zygote pulling” experiment. Individual zygotes were identified microscopically and manipulated away from the mass mating to specific sites on the petri plate. If the zygotes are viable, individual colonies will form. The *wsc1*∆ x *wsc1*∆ zygotes exhibited only ~25% survival rate compared to 96% in *WSC1* x *WSC1* zygotes (*p*-value < 0.01); *WSC1* x *wsc1*∆ zygotes, however, survived at a rate comparable to the wild type crosses (*p*-value > 0.15) (Figure 3A). Given that zygotes form, these data imply that the *wsc1*∆ does not prevent mating, but rather inhibits zygote survival. To examine this further, we used FM4-64 staining and fluorescence microscopy. FM4-64 is a membrane impermeant fluorescent dye that labels the plasma membrane and is used to visualize endocytosis in living yeast [52,53]. In intact cells, FM4-64 initially stains only the plasma membrane, before entering the cell and ultimately being trafficked to the vacuole. As expected, wild-type zygotes restrict FM4-64 to the plasma membrane. However, the cytoplasmic membranes of many *wsc1*∆ x *wsc1*∆ zygotes were brightly stained by FM4-64 (Figure 3B left panels). The bright staining occurred immediately after addition of FM4-64, indicating that the plasma membranes were not intact. The *wsc1*∆ x *wsc1*∆ zygotes showed bright staining ~75% of the time, whereas almost all zygotes with at least one wild type parent showed staining only of the plasma membrane (Figure 3C).

To confirm that the FM4-64 staining of *wsc1*∆ x *wsc1*∆ zygotes did indicate cell death, both Live/Dead and Propidium Iodide staining was performed (Figure 3B). Live/Dead staining relies on FUN 1, a membrane permeable fluorescent dye that allows assessment of metabolic activity in cells [54]. In live cells, FUN 1 accumulates in the vacuoles where it is converted into a red fluorescent molecule. In wild-type zygotes, red fluorescent aggregates were seen within the vacuoles. However, the *wsc1*∆ x *wsc1*∆ zygotes were stained bright green, as expected for dead cells (Figure 3B, central panels). Propidium Iodide is also used to detect cell death [55]. Cells with intact membranes exclude the dye; cells that have lost membrane integrity stain brightly. After incubation with propidium iodide, wild type zygotes showed no fluorescent staining, whereas *wsc1*∆ x *wsc1*∆ zygotes stained brightly (Figure 3B, right panels). These results confirm that zygotes that lack both copies of *WSC1* lose cell integrity and die.

Previous work showed that deletion of *WSC1* causes haploid and homozygous diploid cells to die at high temperatures. In contrast the defect in *wsc1*∆ x *wsc1*∆ mating was observed at normal (30 °C) and low (23 °C) temperatures (Figure S1). The haploid *wsc1*∆ temperature sensitivity can be suppressed by the addition of 1 M sorbitol to the media [39,40]. When matings were performed in the presence of 1 M sorbitol, *wsc1*∆ x *wsc1*∆ zygote death was suppressed (Figure 3D). This observation indicates that the cause of death for the *wsc1*∆ x *wsc1*∆ zygotes is a cell wall defect. This is the first example of *wsc1*∆ associated cell death at normal and low temperatures.

The zygote pulling assay indicated that *wsc1*∆ x *wsc1*∆ zygotes form, but die before they can form a colony. To determine the timing of *wsc1*∆ x *wsc1*∆ zygote death, we used a cytoplasmic transfer assay in conjunction with live-cell imaging. One mating partner was marked with cytoplasmic dsRed, and the other was marked with cytoplasmic GFP (Figure 3E). Imaging of the mating cells showed that death occurred shortly after cytoplasmic transfer, prior to or concurrent with budding. Cell death was apparent by loss of the GFP signal and the formation of dsRed puncta, concomitant with the appearance of large vacuoles evident in the transmitted light images (Figure 3E and Figure S2A). Therefore, cells that lack *WSC1* are able to fuse, but cannot maintain cell wall integrity. Given that death is a property of zygotes, and does not occur prior to cell fusion, we will hereafter refer to the genotype of the dying cells as *wsc1*∆/*wsc1*∆ to indicate their diploid nature.

### 3.2. Overexpression of CWI Pathway Components Suppresses wsc1Δ Zygote Death

**Figure 4:**
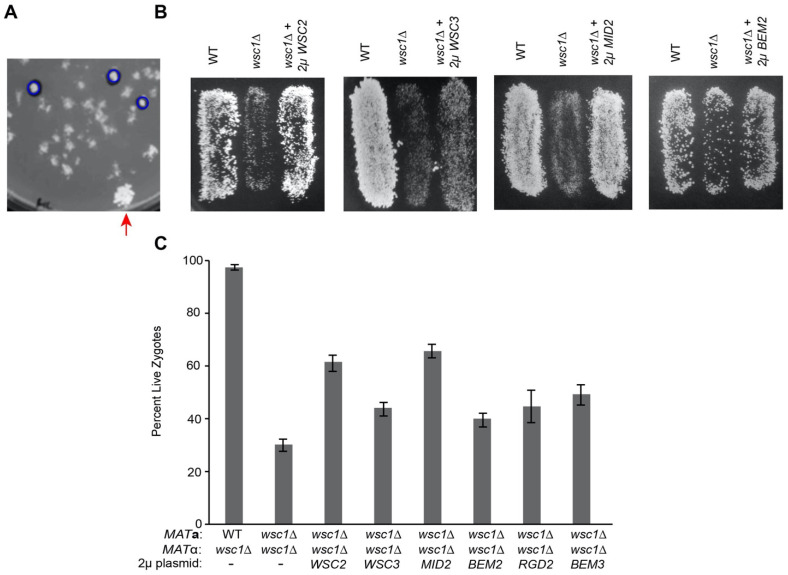
Overexpression of components of the CWI pathway suppress *wsc1*Δ death. (**A**) Example mating plate. A small patch of wild type (MY8093) cells were placed on the mating plate as a control (red arrow). Robust colonies, circled in blue, were picked for retesting. This image is of an 8.5 cm petri dish, the panel is 4.7 cm in width. (**B**) Overexpression suppression of *wsc1*∆. Mating of *WSC2*, *WSC3*, *MID2*, and *BEM2* overexpression in a *wsc1*∆ background. Patches of *MAT*α WT (MY8093) and *wsc1*∆ (MY14306) strains transformed with an empty 2µ vector (pRS425) along with *wsc1*∆ transformed with 2µ *WSC2* (MY15816), 2µ *WSC3* (MY15817), 2µ *MID2* (MR6979) were mated to a *MAT***a** *wsc1*∆ lawn. For *BEM2* overexpression WT (MY8093) and *wsc1*∆ (MY14306) were transformed with empty vector (pRS426) or 2µ *BEM2* (MR6988) and mated as above. Matings were incubated for 3 h at 30 °C and then replica plated onto diploid selective media. In each case the images are of patches of cells growing on 8.5 cm petri dishes; the image panels are 4.0 cm in width. (**C**) Overexpression of potential CWI pathway regulators suppresses *wsc1*∆ x *wsc1*∆ zygote death. WT (MY8092) and *wsc1*∆ (MY14306) were transformed with an empty 2µ vector (pRS425) or *wsc1*∆ was transformed with 2µ *WSC2* (MY15816), *WSC3* (MY15817), *MID2* (MR6979), or *BEM2* (pMR6988). Each *MAT*α was mated to *MAT***a** *wsc1*∆ (MY14305) for 3 h at 30 °C, resuspended in TAF buffer and imaged with FM4-64. n ≥ 100 zygotes for each mating. Error bars represent the standard error. Chi-squared statistical tests were used to calculate significance.

The temperature sensitivity of *wsc1*∆ haploid cells can be suppressed by overexpression of *WSC2*, *WSC3*, *MID2*, *ROM2*, *RHO1*, and *PKC1* [6,9,40]. To determine if the *wsc1*∆/*wsc1*∆ zygote death phenotype was related to the temperature-dependent lysis phenotype, we performed a high-copy suppressor screen using a YEp 13-based yeast genomic library [49]. Colonies of *MAT*α *wsc1*∆ cells containing potential suppressors, along with a control patch of wild type (MY8093) cells, were mated to a *MAT***a** lawn (MY14305) and then replica plated onto diploid selective media. Large colonies, consistent with robust diploid formation, were identified as potential suppressors (Figure 4A). Approximately 24,000 transformants were screened, from which four plasmids were identified which reproducibly and significantly increased diploid formation (*p*-value < 0.01 for each). The genomic inserts on the plasmids contained *WSC1* (2X), *WSC2* (7X), *MID2* (5X), and *BEM2* (5X) (Figure 4B,C). In addition, we also tested high-copy *WSC3*, which provided mild suppression. 

The identification of *WSC2*, *WSC3*, and *MID2* as high-copy suppressors was expected, given that they have been shown to suppress the temperature sensitivity of haploid *wsc1*∆. Suppression by *BEM2*, however, was not expected. *BEM2* acts as a GAP for Rho1p in the CWI pathway, indicating that it should down-regulate the pathway, as opposed to the other suppressors, which would activate the pathway in the absence of *WSC1* [21,23]. However, Bem2p is also known to play a positive role in bud emergence [19,56]; cells that lack Bem2p become large and multinucleate due to a failure to bud [56,57].

Given that overexpression of one Rho-GAP was identified in our screen, we tested the other three *RHO1* GAPS, *SAC7*, *BAG7*, and *LRG1*, as well as *RGD1* and suggested CDC42p GAPS, *RGA1*, *RGA2*, and *RGD2* [5]. In addition to *BEM2*, only *BEM3* and *RGD2* showed relatively modest suppression (*p*-value < 0.03) (Figure 4C). Both Bem3p and Rgd2p have been suggested to act as GAPs for Cdc42p [20,58,59]. Rgd2p has also been shown to act as a GAP for Rho5p, a negative regulator of the CWI pathway [60]. It is likely that the suppression of *wsc1*∆ zygote death by overexpressed Bem2p and Bem3p relates to their regulation of cell wall deposition during budding, whereas suppression by Rgd2p over-expression is due to downregulation of a negative regulator of the CWI pathway. Overexpression of the other Rho-GAP genes did not show any suppression of the *wsc1*∆/*wsc1*∆ zygote formation defect, as seen by plate mating, and were thus not tested further.

### 3.3. Diploid Cells Are Sensitive to Loss of WSC1

**Figure 5:**
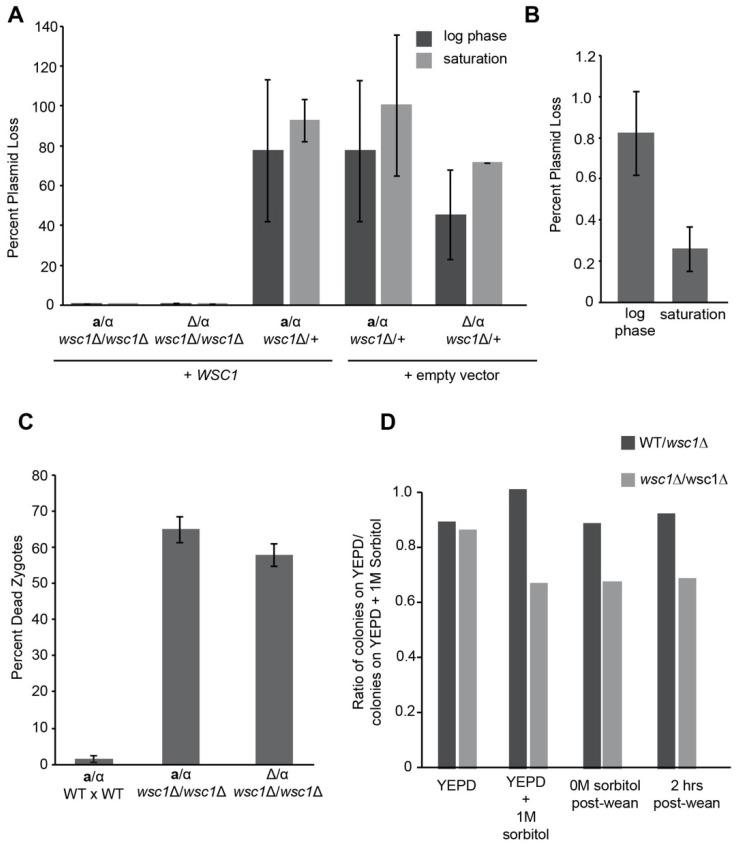
*WSC1* is necessary for diploid growth. (**A**–**C**) *WSC1* dependency is independent of mating type and exacerbated by stationary phase. (**A**) The following strains were transformed with a *URA3* centromeric empty vector plasmid or with the same plasmid bearing *WSC1*: a/α WT/*wsc1*∆ empty vector (MY16050), a/α WT/*wsc1*∆ + *WSC1* (MY16049), a/α *wsc1*∆/*wsc1*∆, ∆/α WT/*wsc1*∆ empty vector (MY16052), ∆/α WT/*wsc1*∆ + *WSC1* (MY16051), and ∆/α *wsc1*∆/*wsc1*∆ + *WSC1* (MY16053). Cells were grown in YEPD liquid log phase (O.D. 0.4–0.7) or saturation and plated on both YEPD plates and 5-FOA plates to quantify plasmid loss. Colonies were counted after two days at 30 °C. (**B**) *WSC1* plasmid loss in a/α *wsc1*∆/*wsc1*∆ during log phase or stationary phase. Experiments were performed three times independently. Error bars represent the standard error of the mean for both A and B. One-tailed Student’s t-test was used to determine significance. (**C**) Filter matings of WT x WT (MY8092 x MY8093), *wsc1*∆ x *wsc1*∆ (MY14305 x MY14306) or *mat*∆ *wsc1*∆ x *MAT*α *wsc1*∆ (MY16045 x MY14306) were performed for 3 h at 30 °C. Cells were resuspended in TAF buffer, stained with FM4-64, and imaged. n ≥ 100 cells for each. Error bars represent the standard error of the mean. Chi-squared statistical tests were used to determine significance. (**D**) *wsc1*∆/wcs1∆ diploid cells adapt to growth without osmotic support. WT/*wsc1*∆ (MY15803) or *wsc1*∆/*wsc1*∆ (MY15805) diploids were grown continuously in YEPD (left most bars) or in YEPD + 1 M sorbitol. The concentration of sorbitol in the media was dropped by 0.2 M every two hours until cells were in 0M sorbitol media when they were plated on YEPD or YEPD + 1 M sorbitol. Cells were allowed to double once without sorbitol and plated as before. Colonies were counted after 2 days at 30 °C and plotted as a ratio of cells on YEPD plates over cells on YEPD + 1 M sorbitol plates. One representative experiment is shown, based on three technical replicates.

Providing a single copy of *WSC1* allows zygotes to survive and form diploids like wild type (Figure S3). We therefore set out to determine whether the increased requirement for Wsc1p occurs only in zygotes or is also a property of mitotic diploid cells. We provided *WSC1* on a low copy, centromeric plasmid, and measured how frequently *wsc1*∆/*wsc1*∆ zygotes lose the plasmid. After maintaining growth in log phase for eight generations, the WT/*wsc1*∆ diploids lost the *WSC1* plasmid over 100-fold more frequently than *wsc1*∆/*wsc1*∆ diploid cells (Figure 5A). *WSC1*/*wsc1*∆ diploids were able to lose the *WSC1* plasmid at a frequency similar to the empty vector (*p*-value = 0.50) (Figure 5A). Thus *wsc1*∆/*wsc1*∆ diploids also show greatly reduced viability, indicating that the defect is not restricted to zygotes. It was previously suggested that *wsc1*∆/*wsc1*∆ diploid cells have a more severe temperature sensitivity when grown to saturation [39]. Consistent with this, the *wsc1*∆/*wsc1*∆ diploid cells maintained in log phase were able to lose the plasmid at a rate 3-fold greater than those allowed to reach saturation (Figure 5B). This is likely an underrepresentation of the actual difference in plasmid loss between log phase and saturated cultures because the saturated cultures have been grown for more generations. 

The greater requirement for *WSC1* in diploid cells could be due to their ploidy or because they are heterozygous at the mating type locus (*MAT*). Previous work suggested that there may be a role for the *MAT* locus in maintaining cell wall integrity [60]. To determine if *wsc1*∆/*wsc1*∆ death was due to diploidy or mating type, we mated a *mat*∆ *wsc*1∆ to a *wsc*1∆ *ΜAΤ*α. The *mat*∆/α is diploid, but, because the *mat*∆ is recessive, the cell exhibits the *MAT*α mating type, like a haploid cell. By both plasmid loss and FM4-64 staining of zygotes, there was no difference between the *MAT***a**/α *wsc1*∆/*wsc1*∆ and the *mat*∆/α *wsc1*∆/*wsc1*∆ strains (Figure 5A,C). Thus, the *wsc1*∆/*wsc1*∆ defect is diploid specific, rather than mating-type specific. 

It is surprising that the severe lysis phenotype of *wsc1*∆/*wsc1*∆ diploids was not previously reported. One possibility is that cells that survive a crisis of growth after *WSC1* plasmid loss are able to adapt to these conditions. To test this, we examined viability as cells were weaned off of osmotic support. *WSC1/wsc1*∆ and *wsc1*∆/*wsc1*∆ diploids that had lost the *WSC1* covering plasmid were selected on media containing 1 M sorbitol. The plasmid-free strains were then grown in either YEPD or in YEPD + 1 M sorbitol. For the cells grown with 1 M sorbitol, the osmolarity of the media was reduced by 0.2 M every two hours to allow one doubling in each condition. Viability was assessed by the ratio of colony forming units on YEPD relative to YEPD + 1 M sorbitol agar plates. The *WSC1*/*wsc1*∆ cells grown in YEPD or YEPD + 1 M sorbitol media had equal viability on either media. When a *wsc1*∆/*wsc1*∆ diploid without the covering plasmid was grown in YEPD, the cells also behaved like the *WSC1*/*wsc1*∆, exhibiting equal viability on YEPD and YEPD + 1 M sorbitol. It is likely that the *wsc1*∆/*wsc1*∆ cells have adapted or accumulated suppressor mutation(s) (Figure 5D). In contrast, the *wsc1*∆/*wsc1*∆ strain that was grown in 1 M sorbitol showed decreased plating efficiency on YEPD, relative to YEPD + 1 M sorbitol. However, the decrease in viability was much less than expected from the frequency of plasmid loss, suggesting that the surviving cells were already largely adapted to the absence of Wsc1p. Cells that were gradually weaned off of osmotic support did not show improved viability on YEPD, relative to the starting culture (Figure 5D), suggesting that adaption takes longer than ~26 generations (generation time includes time to form a colony of FOA plates). FM4-64 staining of the weaned cells showed levels of cell death that were consistent with the reduced viability on YEPD, 27% for *wsc1*∆/*wsc1*∆ versus 14% for *WSC1/wsc1*∆. Taken together, these data suggest that *wsc1*∆/*wsc1*∆ diploid cells adapt, either physiologically or by the accumulation of suppressor mutations.

### 3.4. Overexpression of Hyperactivated Pkc1p Causes Growth Defects

**Figure 6:**
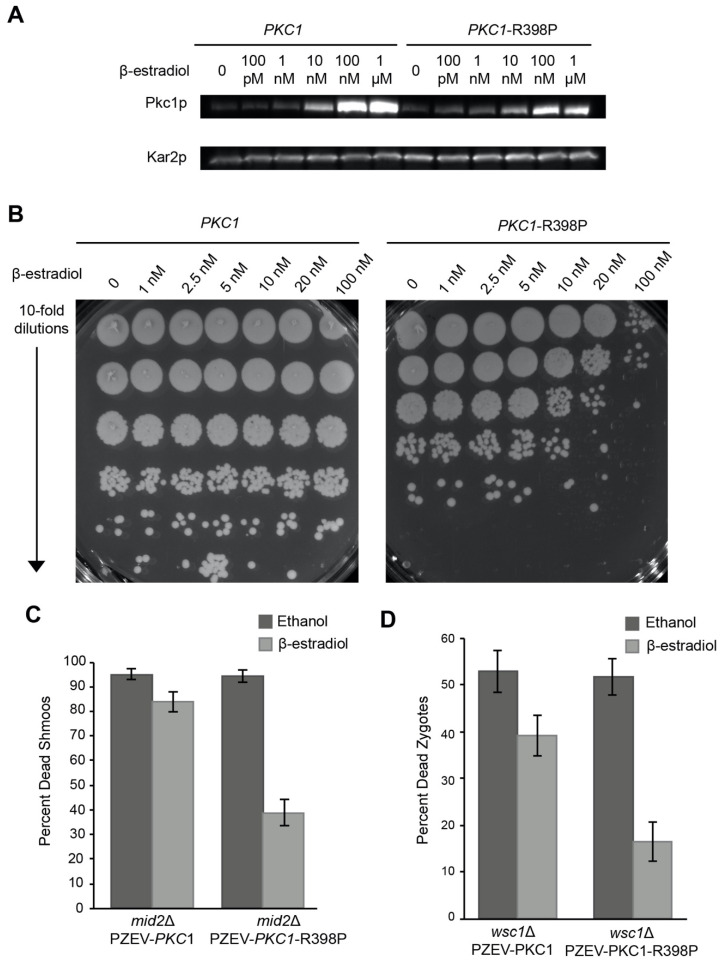
Hyperactivation of *PKC1*-R398P causes growth arrest and suppresses *wsc1*∆ zygote death. (**A**) P_Z3EV_ promoter allows fine control of Pkc1p and Pkc1p^R398P^ protein expression. P_Z3EV_-*PKC1* (MY15622) and P_Z3EV_-*PKC1*-R398P (MY15623) were exposed to varying concentrations of β-estradiol for 30 min. An α-Pkc1p antibody was used to detect protein levels and Kar2p was used as a loading control. (**B**) Hyperactivation of *PKC1*-R398P causes growth arrest. P_Z3EV_-*PKC1* (MY15622) and P_Z3EV_-*PKC1*-R398P (MY15623) strains induced with varying concentrations of β-estradiol for 1.5 h and plated as serial dilutions on YEPD plates. Cells were allowed to grow for 2 days at 30 °C. (**C**) *PKC1*-R398P suppresses *mid2*∆ pheromone-induced death. P_Z3EV_-*PKC1 mid2*∆ (MY15655) and P_Z3EV_-*PKC1*-R398P *mid2*∆ (MY15656) were induced with ethanol or 10 nM β-estradiol and induced with 8 µg/mL α-factor for 3 h and imaged with FM4-64. (**D**) *PKC1*-R398P suppresses *wsc1*∆ zygote death. P_Z3EV_-*PKC1 wsc1*∆ (MY15651 x MY15653) and P_Z3EV_-*PKC1*-R398P *wsc1*∆ (MY15652 x MY15654) were mated in the presence of ethanol or 10 nM β-estradiol and imaged with FM4-64. n ≥ 100 zygotes or shmoos. Error bars represent the standard error. Chi-squared statistical tests were used to determine significance.

Deletion of *PKC1* causes cell lysis at any temperature, which can be suppressed by osmotic support [28,29,30,31]. It has been reported that overexpression of Pkc1p suppresses the temperature sensitivity of *wsc1*∆ haploid cells [6,9,40]. A hyperactive allele of Pkc1p, *PKC1-R398P*, has also been reported to suppress the *mid2*∆ mating-induced death and cause a mild cell fusion defect [6,41]. It was therefore surprising that high copy *PKC1* was not identified in the screen for suppression of *wsc*1∆/*wsc*1∆ zygote death. However, it is possible that high copy *PKC1* is toxic in our conditions or that feedback regulation prevents the over-expressed protein from being sufficiently active. Therefore, we used the PZ_3_EV promoter [61] and β-estradiol induction to express Pkc1p and Pkc1p-R398P under carefully controlled conditions (Figure 6A). To determine the best Pkc1p-R398P expression level, we induced cells with increasing concentrations of β-estradiol and plated serial dilutions to identify the point at which cells failed to grow (Figure 6B). We found that 10 nM β-estradiol was the highest concentration that could be used before significant growth inhibition occurred. At 10 nM a ~10-fold reduction in growth was observed; at 100 nM β-estradiol induction Pkc1p-R398P expression caused ~1000-fold decrease in growth (Figure 6B). In contrast, over-expression of wild-type Pkc1p had no negative effect on cell growth.

*PKC1*-R398P also causes a cell fusion defect, arresting mating cells as prezygotes approximately 16% of the time [41]. Interestingly, we found that high levels of *PKC1-R398P* inhibited the pheromone response in general. Cells containing a galactose inducible *PKC1-R398P* were unable to induce expression of a *FUS1-lacZ* reporter when exposed to α-factor (Figure S5). To determine if activated Pkc1p could suppress *wsc1*Δ we identified a level of Pkc1p-R398P expression that allowed for mitotic growth and a pheromone response. As a positive control for Pkc1p-R398P suppression, we showed that hyperactivated Pkc1p-R398P was able to suppress the *mid2*∆-dependent death of pheromone treated cells (Figure 6C). As the concentration of β-estradiol increased above 1 nM, the efficiency of zygote formation fell rapidly, reaching ~10% of wild-type at 10 nM. A concentration of 10 nM β-estradiol was chosen because this was the lowest concentration at which we saw an increase in PKC1-R398Pp production (Figure 6A), and which also provided some level of mating which would allow measurement of suppression. Among the pre-zygotes that did form, the rate of successful fusion was reduced to ~60%; the remaining 40% were blocked as prezygotes with cell wall remaining between the mating partners (Figure S4A,B). However, consistent with expectation, overexpression of hyper-activated Pkc1p suppressed the death of the *wsc1*∆/*wsc1*∆ zygotes that did form (Figure 6D).

## 4. Discussion

### 4.1. WSC1 Is the Most Important CWI Sensor in Zygotes

*Saccharomyces cerevisiae* cells have a thick cell wall that changes dynamically for growth, mating and to respond to environmental stresses. The CWI pathway is responsible for monitoring the cell wall and ensuring that damage is repaired, or that adequate cell wall deposition is maintained during budding and shmoo formation [4,5]. Of the five transmembrane proteins that regulate the CWI pathway, *WSC1* and *MID2* are the most important; only *wsc1* and *mid2* mutants exhibit phenotypes, and cells containing only *WSC1* and *MID2* appear to be wild type [10]. Through this and previous work, it is apparent that *WSC1* and *MID2* play different roles; Mid2p is important during shmoo formation, whereas Wsc1p is important for maintaining cell wall integrity during mitotic growth and post fusion [10]. Deletion of *WSC1* was previously shown to cause cell lysis at high temperatures in both haploids and diploids [32,39,40]. However, using a mating assay we demonstrated that *WSC1* is actually required at all temperatures in diploids, and that previous work underestimated its role. 

The *wsc1*∆ defect was initially observed during mating, as a strong zygote specific phenotype. Why might zygotes be different from haploid cells? One of the striking differences between them is their shape. Since *MID2* plays a large role in shmoos, but not in zygotes, it seems likely that Wsc1p acts as the major cell wall sensor in zygotes. However, using a plasmid loss assay, it is apparent that diploid cells also require *WSC1* to survive in log phase, and this phenotype is exacerbated when cells reach saturation. Haploid cells lacking *WSC1* have a slightly decreased growth rate, but are otherwise comparable to wild type. Why, then, would zygotes and diploid cells die without *WSC1*? Both zygotes and diploids are different in shape than haploid cells. Diploid cells are more ovoid and are twice the volume of haploid cells, on average [62]. Although, it has been shown that *MAT***a**/α diploid cells down regulate components of the cell wall synthesis machinery [62], which should exacerbate the defect of cells lacking *WSC1*. However, heterozygosity at the mating type locus is not sufficient to explain the defect, as *mat*∆/α still require Wsc1p. Another difference between haploid and diploid cells is their surface area to volume ratio; diploids have a lower surface area to volume ratio than haploid cells. It is possible that this accounts for the increased need for *WSC1* in diploids and is independent of ploidy. Interestingly, diploid cells can adapt to growth without *WSC1*. Although *wsc1*∆/*wsc1*∆ cells can lose the *WSC1* covering plasmid at a frequency of less than 1% (Figure 5B), diploids can survive when grown with osmotic support. The surviving *wsc1*∆/*wsc1*∆ diploids are eventually able to grow without Wsc1p, either through adaptation or suppressor accumulation (Figure 5D). Perhaps *WSC2* or *WSC3*, both of which can suppress the zygote death of *wsc1*∆ when overexpressed, are upregulated to account for the loss of the key CWI sensor.

### 4.2. WSC1 May Be Involved in Maintaining Cell Wall Integrity during Bud Emergence

Unsurprisingly, overexpression of components of the CWI pathway suppress *wsc1*∆ zygote death. Overexpression of *BEM2*, *BEM3*, and *RGD2* were also able to suppress, which was surprising given that other GAPs for Rho1p in the CWI pathway did not. The *wsc1*∆ cells did not lyse until after fusion, and generally as a zygote with a small bud (Figure 3E and Figure S2A). Whereas suppression by positive regulators of the CWI pathway are likely to suppress by bypassing the need for the upstream sensor, it seems unlikely that *BEM2*, *BEM3*, and *RGD2* function in a similar manner. Overexpression of *BEM2* should downregulate the CWI pathway and exacerbate the *wsc1*∆ defect. Both *BEM2* and *BEM3*, however, have been shown to play a role in bud emergence that may be independent of their function as GAPs. For example, Bem3p is a GAP for Cdc42p [19,56,63]. However, it seems unlikely that suppression by *BEM3* overexpression is through its Cdc42p GAP activity because overexpression of *RGA1* and *RGA2*, the other GAPS for Cdc42p do not suppress. In the absence of *WSC1* perhaps cells form zygotes, but are unable to regulate cell wall deposition to produce viable buds. It is also possible that *BEM2* and *BEM3* are acting as effectors rather than GAPs to suppress the zygote death phenotype. GAP proteins are generally thought to increase hydrolysis of GTP, thus inactivating the G-protein they interact with [64,65]. There is evidence, however, that GAPs act as effectors as well, potentially having a positive regulatory role downstream of the G-proteins they act upon [66]. *BEM2* may act as an effector for Rho1p in this case, activating something downstream in the CWI pathway and allowing for proper cell wall deposition and zygote survival. Suppression of the *wsc1*∆ defect must occur either though decreased cell wall removal or increased cell wall deposition, allowing for zygote survival. Though less likely, it is also possible that *BEM3* downregulation of Cdc42p activity could lead to decreased cell wall removal during budding and subsequent diploid growth, thereby suppressing the defect.

*RGD2* is less well studied, but is known to act as a GAP for both Rho5p and Cdc42p [20,58,59,60]. Deletion of Rho5p leads to resistance to drugs such as caffeine, suggesting that its role is to down regulate the CWI pathway [60]. As a GAP, Rgd2p downregulates Rho5p activity, and would potentially lead to increased CWI pathway signaling. Thus, *RGD2* overexpression likely suppresses in a manner similar to *WSC2*, *WSC3*, and *MID2*. The fact that we did not identify specific components of the CWI pathway downstream of Rho1p and Pkc1p suggests that it is the activity of these two proteins that allows for proper cell wall deposition, rather than downstream signaling via the MAP kinase cascade. For example, Rho1p’s role in regulating glucan synthase may be important for overcoming defects in the upstream components of the CWI pathway.

### 4.3. Constitutive Activation of the CWI Pathway Is Detrimental to Cells

Previous work showed that a hyperactive allele of *PKC1* (*PKC1-R398P*) causes a block in cell fusion, implicating the CWI pathway as a negative regulator of fusion [41]. Pkc1p acts downstream of *WSC1* in the CWI pathway. Surprisingly, we found that Pkc1p-R398P overexpression is toxic, leading to growth arrest and an inability to respond to pheromone (Figure 6B and Figure S5). It is likely that over-activation of Pkc1p signals constant cell wall damage that must be repaired. Pkc1p responds to cell wall damage in part by relocalizing to the site of damage [67]. Perhaps, overexpression of hyperactive Pkc1p leads to mislocalization over the entire cell cortex, signaling cell wall damage everywhere, and blocking the cell wall remodeling required for growth. Alternatively, given that the signaling output from the CWI pathway impinges on cell cycle regulation, a constant signal from Pkc1p might cause cells to arrest [5]. However, given moderate amounts of Pkc1p activity, cells can overcome the loss of either *MID2* or *WSC1*. 

Although the complexity of the downstream signaling of the CWI pathway has been understood for some time, the specific functions of the transmembrane components is surprisingly complex. *MID2* and *WSC1* are the key cell wall sensors, but perform different functions in sensing the state of the cell wall. *MID2* is the primary sensor during shmoo formation, whereas *WSC1* plays a surprisingly crucial role in zygote and diploid survival, beyond that known for haploid cell growth. 

## Data Availability

The data presented in this study are available in the main body of text or the supplementary material. All strains are available upon request.

## Supplementary Materials

The following are available at https://www.mdpi.com/article/10.3390/jof7121049/s1. Figure S1—*wsc1*Δ/*wsc1*Δ show diploid formation defects at 23 °C. Figure S2—*wsc1*Δ/*wsc1*Δ zygote die after fusion. Figure S3—*WSC1* covering plasmid supports diploid formation. Figure S4—Induction of PZ3EV-Pkc1pR398P causes zygote formation and fusion defects. Figure S5—Hyperactivation of *PKC1-R398P* inhibits the pheromone response. Table S1—Yeast Strains. Table S2—Plasmids.
