# Supplementary material for: Differential Requirement for the Cell Wall Integrity Sensor Wsc1p in Diploids Versus Haploids
Source: J Fungi (Basel). 2021 Dec 8;7(12):1049. doi: 10.3390/jof7121049 (PMC8703914; doi:10.3390/jof7121049)
Supplement: Supplementary file 1 [file jof-07-01049-s001.zip › jof-1484699-supplementary.pdf]

Supplemental Materials and Methods

Strains induced with galactose were grown overnight, diluted and allowed to grow to early log phase in media containing 2% raffinose. Cells were then treated with 2% galactose concurrent with pheromone induction for the times specified. β-Galactosidase assays to measure induction of the mating pathway were performed on log phase, pheromone-induced cultures as described [64].

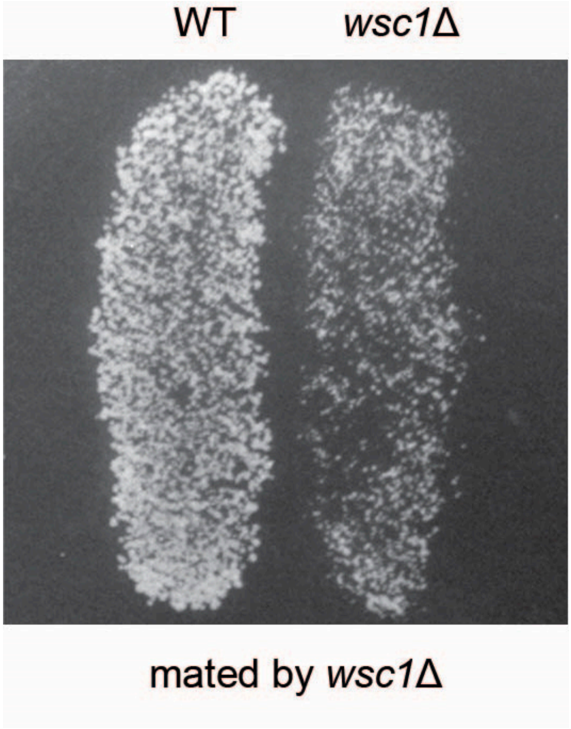

Figure S1: *wsc1*Δ/*wsc1*Δ show diploid formation defects at 23ºC.

WT (MY8092) or *wsc1*Δ (MY14305) *MAT***a** stains mated to a *wsc1*Δ *MAT*α (MY14306) lawn for 3 hours at 23°C and replica plated onto diploid selective media.

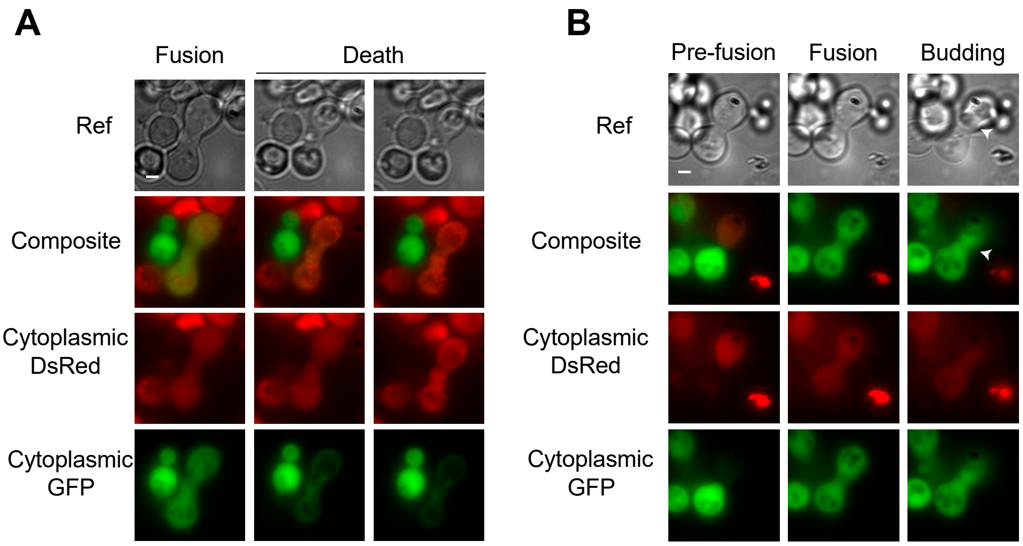

Figure S2: *wsc1*Δ/*wsc1*Δ zygotes die after fusion.

(A) Live imaging of *wsc1*Δ x *wsc1*Δ mating. *wsc1*Δ *MAT***a** (MY15101) cells transformed with an integrating plasmid containing cytoplasmic GFP (MR5909) were mated to *wsc1*Δ *MAT*α (MY15102) cells transformed with an integrating plasmid containing cytoplasmic DsRed (MR5908). Cells were mixed and imaged at 2 minute intervals. Images show cells after fusion when both markers have transferred, and the change in fluorescence as zygotes die, specifically the loss of GFP fluorescence. **(B)** Live imaging of a living zygote. Images show cells pre-fusion, before the cytoplasmic markers transfer, after fusion has occurred, and as the zygote begins to bud (white arrow head). The GFP signal persists even as budding occurs in the living zygotes. Part of the zygote moves out of view as it begins to bud. Scale bar = 2μ

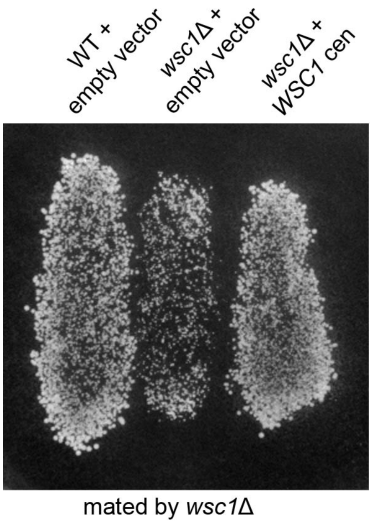

Figure S3: *WSC1* centromeric covering plasmid supports diploid formation.

A WT (MY8093) strain was transformed with an empty *URA3* centromeric plasmid (pRS416) and a *wsc1*Δ (MY14306) strain was transformed with either the empty *URA3* centromeric plasmid or a *WSC1 URA3* centromeric plasmid (MR7028). The *MAT*α stains were mated to a *MAT***a** *wsc1*Δ (MY14305) lawn for 3 hours at 30°C and then replica plated onto diploid selective media. Plates were allowed to incubate for two days at 30°C.

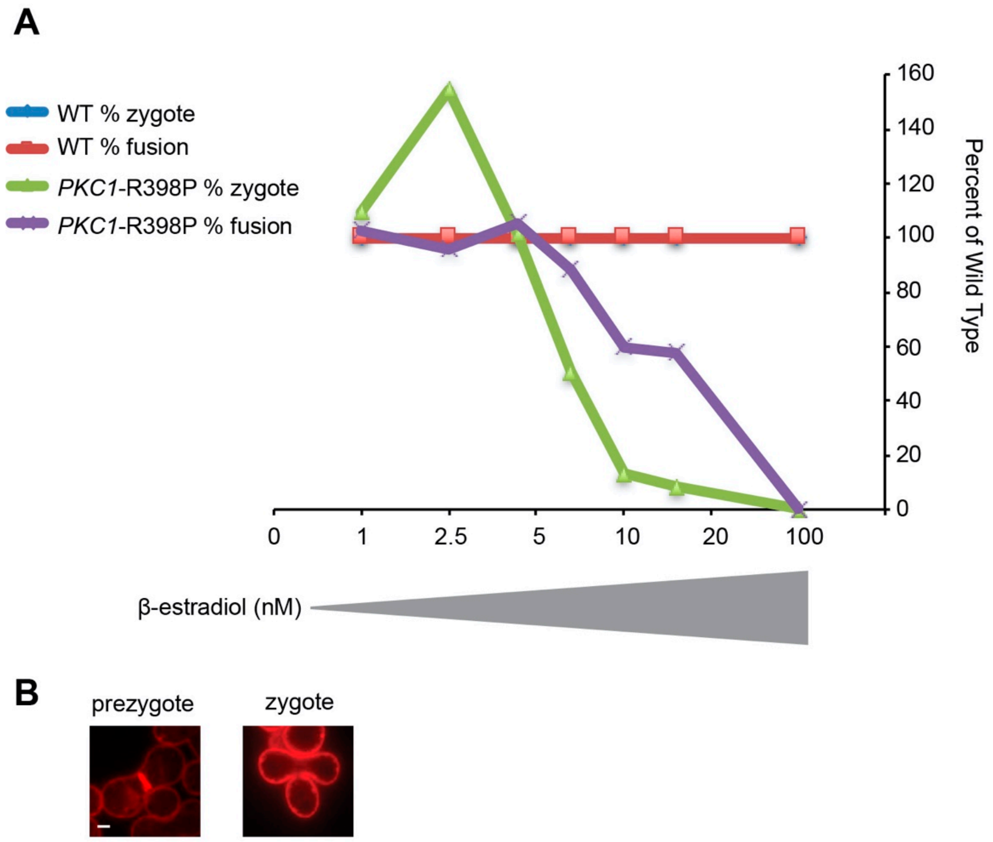

Figure S4: Induction of P_{Z3EV}-Pkc1p^{R398P} causes zygote formation and fusion defects.

(A) A representative experiment showing varying concentrations of β-estradiol allow assessment of *PKC1-R398P* fusion defect. Mating of P_{Z3EV}-*PKC1* (MY15622 x MY15624) and P_{Z3EV}-*PKC1-R398P* (MY15623x MY15625) strains were performed after 3-hour induction with β-estradiol at varying concentrations. Percent of cells that were able to form zygotes and percent fusion was normalized to the wild type mating. More zygotes were formed in the single experiment shown at 2.5 nM β-estradiol, in the P_{Z3EV}-*PKC1-R398P* mating than wild type. **(B)** Representative images of a prezygote and zygote with a large bud formed using 10nM β-estradiol and *PKC1-R398P*. Scale bar = 2μ.

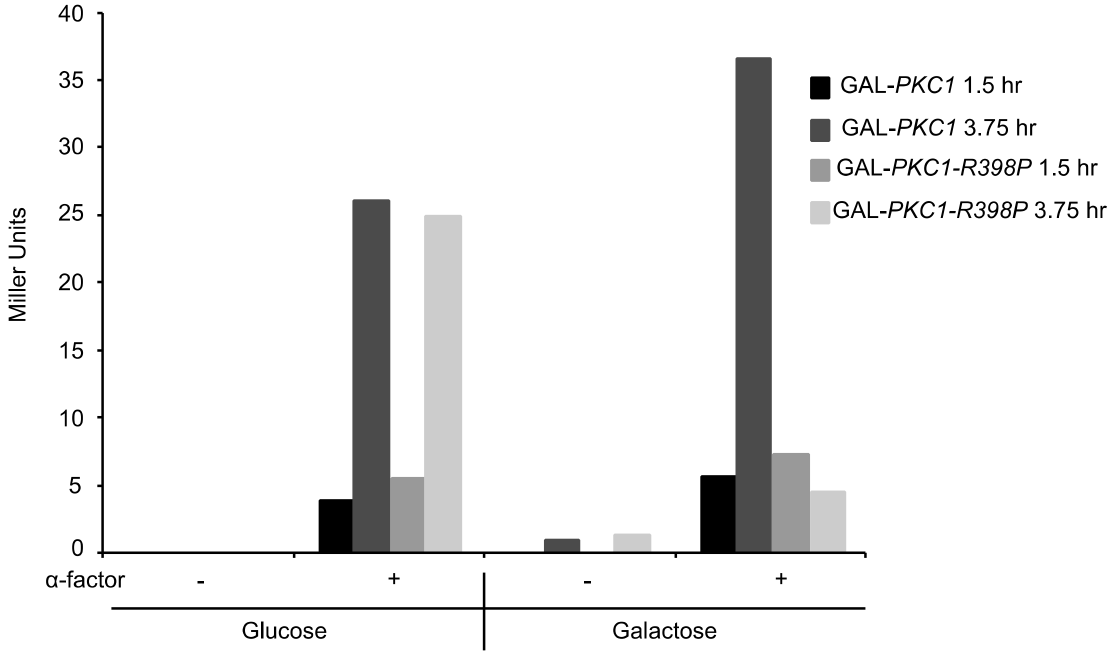

Figure S5: Hyperactivation of *PKC1-R398P* inhibits pheromone response.

A WT (MY8092) stain was transformed with a *FUS1*-lacZ plasmid (MR7010) and either a *GAL-PKC1* plasmid (pDL298) or *GAL-PKC1*-R398P plasmid (pDL242). These strains were either treated with 2% galactose or 2% glucose and with and without pheromone (pheromone and sugar were added at the same time) for 1.5 hours or 3.75 hours prior to the β-galactosidase measurement.

Table S1. **Yeast strains**

**Strain**	**Genotype**	**Source**
JY429	*MATα fus1-Δ1 fus2- Δ3 trp1- Δ1 ura3-52 cyh2*	G. Fink (Whitehead Institute, Cambridge, MA)
MY8092	*MAT***a** *his3Δ1 leu2Δ0 ura3Δ0 met15Δ0*	
MY8093	*MATα his3Δ1 leu2Δ0 ura3Δ0 lys2Δ0*	
MY14305	*MAT***a** *wsc1::KANMX his3Δ1 leu2Δ0 ura3Δ0 met15Δ0*	
MY14306	*MATα wsc1::KANMX his3Δ1 leu2Δ0 ura3Δ0 lys2Δ0*	
MY14673	*MAT***a** *mid2::HIS3 his2Δ1 leu2Δ0 ura3Δ0 met15Δ0*	
MY14674	*MATα mid2::HIS3 his2Δ1 leu2Δ0 ura3Δ0 lys2Δ0*	
MY15101	*MAT***a** *wsc1::KANMX SSO1::*P^{GPD1}*-GFP::URA3 his3Δ1 leu2Δ0 ura3Δ0 met15Δ0*	
MY15102	*MATα wsc1::KANMX SSO1::*P^{GPD1}*-dsRED::URA3 his3Δ1 leu2Δ0 ura3Δ0 lys2Δ0*	
MY15622	*MAT***a** *can1::*P_{Z3EV}*PKC1 leu2Δ0::*P_{ACT1}*-*Z_3EV*-NATMX ura3Δ0*	
MY15623	*MAT***a** *can1::*P_{Z3EV}*PKC1-R398P leu2Δ0::*P_{ACT1}*-*Z_3EV*-NATMX ura3Δ0*	
MY15624	*MATα can1::*P_{Z3EV}*PKC1 leu2Δ0::*P_{ACT1}*-*Z_3EV*-NATMX ura3Δ0*	
MY15625	*MATα can1::*P_{Z3EV}*PKC1-R398P leu2Δ0::*P_{ACT1}*-*Z_3EV*-*	

	NATMX ura3Δ0
MY15651	*MAT***a** *wsc1::KANMX can1::*P_{Z3EV}*PKC1 leu2Δ0::*P_{ACT1}*-*Z_3*EV-NATMX ura3Δ0*
MY15652	*MAT***a** *wsc1::KANMX can1::*P_{Z3EV}*PKC1_R398P leu2Δ0::*P_{ACT1}*-*Z_3*EV-NATMX ura3Δ0*
MY15653	*MAT*α *wsc1::KANMX can1::*P_{Z3EV}*PKC1 leu2Δ0::*P_{ACT1}*-*Z_3*EV-NATMX ura3Δ0*
MY15654	*MAT*α *wsc1::KANMX can1::*P_{Z3EV}*PKC1-R398P leu2Δ0::*P_{ACT1}*-*Z_3*EV-NATMX ura3Δ0*
MY15655	*MAT***a** *mid2::KANMX can1::*P_{Z3EV}*PKC1 leu2Δ0::*P_{ACT1}*-*Z_3*EV-NATMX ura3Δ0*
MY15656	*MAT***a** *mid2::KANMX can1::*P_{Z3EV}*PKC1-R398P leu2Δ0::*P_{ACT1}*-*Z_3*EV-NATMX ura3Δ0*
MY15803	*MAT***a**/*MATα wsc1::KANMX/WSC1his3Δ1/ his3Δ1 leu2Δ0 / leu2Δ0 ura3Δ0/ ura3Δ0*
MY15805	*MAT***a**/*MATα wsc1::KANMX/wsc1::KANMX his3Δ1/ his3Δ1 leu2Δ0 / leu2Δ0 ura3Δ0/ ura3Δ0*
MY15816	*MAT***a** *his3Δ1 leu2Δ0 ura3Δ0 met15Δ0 WSC2-LEU-2μ*
MY15817	*MAT***a** *his3Δ1 leu2Δ0 ura3Δ0 met15Δ0 WSC3-LEU-2μ*

MY16045	*matΔ::leu2 wsc1::KANMX ura3-52 leu2-3 trp1Δ1 his3::TRP1*
MY16049	*MAT***a***/MATα wsc1::KANMX/WSC1 his3Δ1/ his3Δ1 leu2Δ0 / leu2Δ0 leu2Δ0/ leu2Δ0 ura3Δ0/ ura3Δ0 WSC1-URA3*
MY16050	*MAT***a***/MATα wsc1::KANMX/WSC1 his3Δ1/ his3Δ1 leu2Δ0 / leu2Δ0 ura3Δ0/ ura3Δ0*
MY16051	*MAT***a***/MATα wsc1::KANMX/wsc1::KANMX his3Δ1/ his3Δ1 leu2Δ0 / leu2Δ0 ura3Δ0/ ura3Δ0 WSC1-URA3*
MY16052	*MATΔ/MATα wsc1::KANMX/wsc1::KANMX his3Δ1/ his3Δ1 leu2Δ0 / leu2Δ0 ura3Δ0/ ura3Δ0*
MY16053	*MATΔ/MATα wsc1::KANMX/wsc1::KANMX his3Δ1/ his3Δ1 leu2Δ0 / leu2Δ0 ura3Δ0/ ura3Δ0 WSC1-URA3*

Table S2. **Plasmids**

**Strain**	**Genotype**	**Reference**
pMR5908	*URA3 SSOL' PGPD1-dsRED* amp^R	(Jin et al., 2004)
pMR5909	*URA3 SSOL' PGPD1-GFP* amp^R	(Jin et al., 2004)
pMR6979	*MID2 LEU2* 2μ amp^R	
pMR6988	*BEM2* URA3 2μ amp^R	
pMR7010	FUS1-lacZ LEU2 CEN4 ARS1 ampR	
pMR7028	*WSC1 URA3 CEN3 ARS1* amp^R	
pRS415	*LEU2 CEN3 ARS1* amp^R	(Sikorski and Hieter, 1989)
pRS416	*URA3 CEN3 ARS1* amp^R	(Sikorski and Hieter, 1989)
pRS425	LEU2 2μ amp^R	(Sikorski and Hieter, 1989)
pRS426	URA3 2μ amp^R	(Sikorski and Hieter, 1989)

pDL242	*pGAL[PKC1-R398A] URA3 CEN3 amp*^{R}	(Philips and Herskowitz, 1997)
pDL293	*pGAL[PKC1::HA] URA3 CEN3 amp*^{R}	(Philips and Herskowitz, 1997)
